# Prolonged ex-vivo normothermic kidney perfusion: The impact of perfusate composition

**DOI:** 10.1371/journal.pone.0251595

**Published:** 2021-05-18

**Authors:** Merel B. F. Pool, Tim L. Hamelink, Harry van Goor, Marius C. van den Heuvel, Henri G. D. Leuvenink, Cyril Moers

**Affiliations:** 1 Department of Surgery–Organ Donation and Transplantation, University Medical Center Groningen, University of Groningen, Groningen, The Netherlands; 2 Department of Pathology and Medical Biology, University Medical Center Groningen, University of Groningen, Groningen, The Netherlands; University Medical Center Utrecht, NETHERLANDS

## Abstract

Normothermic machine perfusion (NMP) of donor kidneys provides the opportunity for improved graft preservation and objective pre-transplant ex-vivo organ assessment. Currently, a multitude of perfusion solutions exist for renal NMP. This study aimed to evaluate four different perfusion solutions side-by-side and determine the influence of different perfusate compositions on measured renal perfusion parameters. Porcine kidneys and blood were obtained from a slaughterhouse. Kidneys underwent NMP at 37°C for 7 hours, with 4 different perfusion solutions (n = 5 per group). Group 1 consisted of red blood cells (RBCs) and a perfusion solution based on Williams’ Medium E. Group 2 consisted of RBCs, albumin and a balanced electrolyte composition. Group 3 contained RBCs and a medium based on a British clinical NMP solution. Group 4 contained RBCs and a medium used in 24-hour perfusion experiments. NMP flow patterns for solutions 1 and 2 were similar, solutions 3 and 4 showed lower but more stable flow rates. Thiobarbituric acid reactive substances were significantly higher in solution 1 and 4 compared to the other groups. Levels of injury marker N-acetyl-β-D glucosaminidase were significantly lower in solution 2 in comparison with solution 3 and 4. This study illustrates that the perfusate composition during NMP significantly impacts the measured perfusion and injury parameters and thus affects the interpretation of potential viability markers. Further research is required to investigate the individual influences of principal perfusate components to determine the most optimal conditions during NMP and eventually develop universal organ assessment criteria.

## Introduction

Worldwide, the standard donor kidney preservation method is static cold storage (SCS). However, in the Netherlands hypothermic machine perfusion (HMP) is clinically used to preserve all deceased donor kidneys. HMP showed a decrease in delayed graft function and an improved 1- and 3-year renal graft survival in comparison with SCS preservation [1,2]. In order to minimise the substantial gap between the supply of and demand for donor kidneys, suboptimal deceased-donor kidneys are increasingly being used. Due to the increased use of these less than optimal quality donor kidneys, it is of the utmost importance to establish even further optimised strategies for robust and objective pre-transplant assessment as well as preservation. The use of normothermic machine perfusion (NMP) for these deceased-donor kidneys is increasingly being considered. Pre-clinical studies have shown that NMP may lead to a better transplant outcome compared to SCS alone [3]. As donation after cardiac death and extended criteria donor kidneys are less tolerant of hypothermic storage compared to organs derived from standard criteria donor procedures, marginal-quality donor organs could benefit from NMP preservation [4,5]. Currently, only one clinical renal NMP trial is being conducted in the UK, with a relatively short pre-transplant perfusion period of 1 hour [6]. For adequate organ assessment and resuscitation, however, longer NMP times are conceivably necessary [7]. This method provides the opportunity for pre-transplant organ diagnostics, improved preservation, and ex-vivo interventions prior to transplantation to improve post-transplant renal function. Nowadays, many transplant centres have started to invest in this promising normothermic ex-vivo perfusion strategy. Among these centres, a wide variety of NMP perfusion solutions exists. The large heterogeneity in composition could impede the ultimate goal to define uniform and robust NMP assessment criteria. This heterogeneity is partly driven by the currently only limited understanding of what the exact formulation of an NMP solution should be. Most likely, a normothermic perfusion solution has to contain an oxygen carrier, a colloid, and a balanced electrolyte composition [8]. The composition will also depend on the duration of NMP, as longer periods of perfusion will require optimised perfusate compositions to maintain a stable near-physiological environment throughout the entire perfusion. This study evaluated four different normothermic perfusion solutions, three commonly used existing solutions to which no alterations were made and one newly designed solution. We conducted prolonged (7h) NMP in a porcine kidney donation model. Our aim was to analyse to what extent different perfusate compositions as a whole impact electrolyte balance, renal function, and injury markers measured during NMP.

## Materials and methods

### Kidney and blood retrieval

Viable porcine kidneys from domestic landrace pigs (sow; type Topigs 20) and blood were obtained from a local slaughterhouse (Kroon Vlees, Groningen, the Netherlands). Pigs were stunned with a bi-temporal electric shock and subsequently exsanguinated according to normal slaughterhouse procedures. Autologous blood was collected and heparin (5000 international units per ml (IU), LEO® pharma, Ballerup, Denmark) was added to prevent blood from clotting. Kidneys were retrieved rapidly after circulatory death of the pig, resulting in approximately 20 minutes of warm ischaemia (WI) before cold preservation. After the WI period, kidneys were flushed and cooled with 180 ml sodium chloride (NaCl) (0.9%) (Baxter B.V., Utrecht, the Netherlands) at 4°C, which marked the start of cold ischaemia (CI). Kidneys were dissected free of excess fatty tissue and blood vessels were exposed. Next, kidneys were connected to a Kidney Assist Transport HMP device (Organ Assist, Groningen, the Netherlands). This HMP machine preserved kidneys with cold (0–4°C) UW machine perfusion solution (Belzer UW-MP solution, Bridge to Life Ltd, Columbia, SC) for 2–4 hours using oxygenated pulsatile HMP at a mean arterial pressure of 25 mmHg. Heparinised autologous blood was leukocyte depleted using a leukocyte filter (BioR 02 plus BS PF, Fresenius Kabi, Zeist, the Netherlands), centrifuged and subsequently, supernatant plasma was removed.

### Perfusion solutions

Four experimental groups were defined (n = 5 per group), with a different NMP solution in each group. Prior to kidney retrieval, the perfusion solution which would be used during the experiment was determined. All four perfusion solutions contained autologous porcine red blood cells (RBCs), but the composition of each NMP medium was different (Table 1).

**Table 1:** Composition of the four perfusion solutions.

Group 1	Group 2	Group 3	Group 4
**350 ml autologous RBCs**	**350 ml autologous RBCs**	**170 ml autologous RBCs**	**290 ml autologous RBCs**
**40 g bovine serum albumin** (Bovine Serum Albumin fraction V, Roche, Mannheim, Germany)	**200 ml Albuman 200 g/l** (Sanquin Plasma Products B. V., Amsterdam, Netherlands)	**120 ml saline, adenine, glucose, mannitol (SAG-M)** (also Sanquin)	**100 ml Albuman 200 g/l** (also Sanquin Plasma Products B. V.)
**500ml Williams’ Medium E** (Gibco^®^ William’s Medium E + GlutaMAX™, Life Technologies Ltd, Bleiswijk, Netherlands)	**250 ml NaCl 0,9%** (Fresenius Kabi Nederland B.V., Zeist, Netherlands)	**290 ml Ringers lactate** (Baxter B.V., Utrecht, Netherlands)	**300 ml NaCl 0.9%** (also Fresenius Kabi Nederland B.V.)
**10 ml Augmentin** (1200mg/10 ml) (Sandoz B.V., Almere, Netherlands)	**2 ml Augmentin** (1200 mg/10ml) (also Sandoz BV)	**2 ml Augmentin** (1200 mg/10ml) (also Sandoz BV)	**8 ml Augmentin** (1200 mg/10ml) (also Sandoz BV)
**1000 μmol/l creatinine** (Sigma-Aldrich, Zwijndrecht, Netherlands)	**1000 μmol/l creatinine** (also Sigma-Aldrich)	**1000 μmol/l creatinine** (also Sigma-Aldrich)	**1000 μmol/l creatinine** (also Sigma-Aldrich)
	**21 ml NaHCO**_**3**_ **8,4%** (B. Braun Melsungen AG, Melsungen, Germany)	**27 ml NaHCO**_**3**_ **8,4%** (also B. Braun)	**8 ml NaHCO**_**3**_ **8,4%** (also B. Braun)
	**1,4 ml MgSO**_**4**_ **100 mg/ml** (0,4 mmol/ml) (Teva Nederland B.V., Haarlem, Netherlands)	**10 ml mannitol 15%** (also Baxter B.V.)	**10 mg mannitol** (also Baxter B.V.)
	**6,5 ml calcium gluconate** (0,225 mmol/ml) (also B. Braun)	**3 ml heparin** (also LEO® pharma)	**4,8 ml calcium gluconate** (0,225 mmol/ml) (also B. Braun)
	**11 ml glucose 5%** (also Baxter B.V.)	**3,75 mg dexamethasone** (also Centrafarm)	**9,6 ml glucose 5%** (also Baxter B.V.)
	**2,5 ml KCl 74,6 mg/ml** (1 mmol/ml) (Centrafarm BV, Etten-Leur, Netherlands)		**8 IU insulin** (Novo Nordisk A/S, Bagsværd, Denmark)
	**0,2 mL Na**_**3**_**PO**_**4**_ (3 mmol/ml) (Apotheek A15, Gorinchem, Netherlands)		
	**90 ml sterile water** (also Fresenius Kabi)		

In our laboratory, ample experience has been gained with the perfusion of porcine kidneys and rodent livers using Williams’ Medium E (WME) (Life Technologies Ltd, Bleiswijk, Netherlands) [9–11]. This perfusate does not contain a vasodilator and we decided not to make alterations to the initial solution. The perfusion solution of group 2 was developed by our group to contain electrolytes in physiological concentrations and exert a physiological colloid osmotic pressure on the glomerular membrane. The group 3 perfusate was a colloid-free clinically used British NMP solution, which is currently utilised in a randomised controlled trial comparing 1h NMP with SCS of human deceased donor kidneys in the United Kingdom [12]. As for group 4, this solution was successfully used in a porcine autotransplantation study in Aarhus, Denmark [13]. It is based on the perfusate designed by Weissenbacher et al., who perfused discarded human kidneys during 24 hours [7].

In addition to the above-mentioned constituents, the perfusate in group 1 was supplemented with glucose when concentration dropped below 4 mmol/l. Produced urine was hourly replaced with WME. In group 2 a syringe infusion pump infused total parenteral nutrition (SmofKabiven) (Fresenius Kabi Nederland B.V., Zeist, Netherlands) at a rate of 0.5 ml/h and insulin (1000 IU, also Novo Nordisk A/S) at a rate of 0.005 ml/h. After every hour, urine production was recirculated in group 2 to maintain a stable electrolyte balance.

In the third group, 170 ml pure RBCs were mixed with 120 ml saline, adenine, glucose and mannitol (SAG-M), leading to a haematocrit of 0.5–0.65 l/l, to mimic the composition of a typical clinically used unit of RBCs (which is utilised in the British clinical NMP perfusate). During these experiments, three syringe infusion pumps were used to infuse Flolan (GlaxoSmithKline B.V., Zeist, Netherlands) at a rate of 5 ml/h, glucose 5% (Baxter B.V., Utrecht, Netherlands) at a rate of 4 ml/h and a mixture of 150 ml of Synthamin-17 10% (Baxter Healthcare Ltd., Norfolk, United Kingdom), 6 ml sodium bicarbonate (NaHCO_3_) 8.4% (B. Braun Melsungen AG, Melsungen, Germany), 30 IU insulin (1000 IU, Novo Nordisk A/S, Bagsværd, Denmark) and Cernevit (Baxter B.V., Utrecht, Netherlands) at a rate of 20 ml/h, according to the protocol of the current clinical UK study. Urine production was hourly replaced by Ringers lactate (also Baxter B.V.).

During NMP of the fourth group, a syringe infusion pump infused verapamil (2.5 mg/ml, also Sandoz B.V.) dissolved in saline at a rate of 0.3 ml/h. Urine production was also recirculated in this group after every hour to maintain a stable electrolyte balance.

Sample volumes in each group were corrected with different components. The volume in group 1 was replaced with WME, in group 2 with perfusion medium, in group 3 with Ringers lactate and in group 4 with Sterofundin® (also B. Braun).

### NMP setup

**Fig 1:**
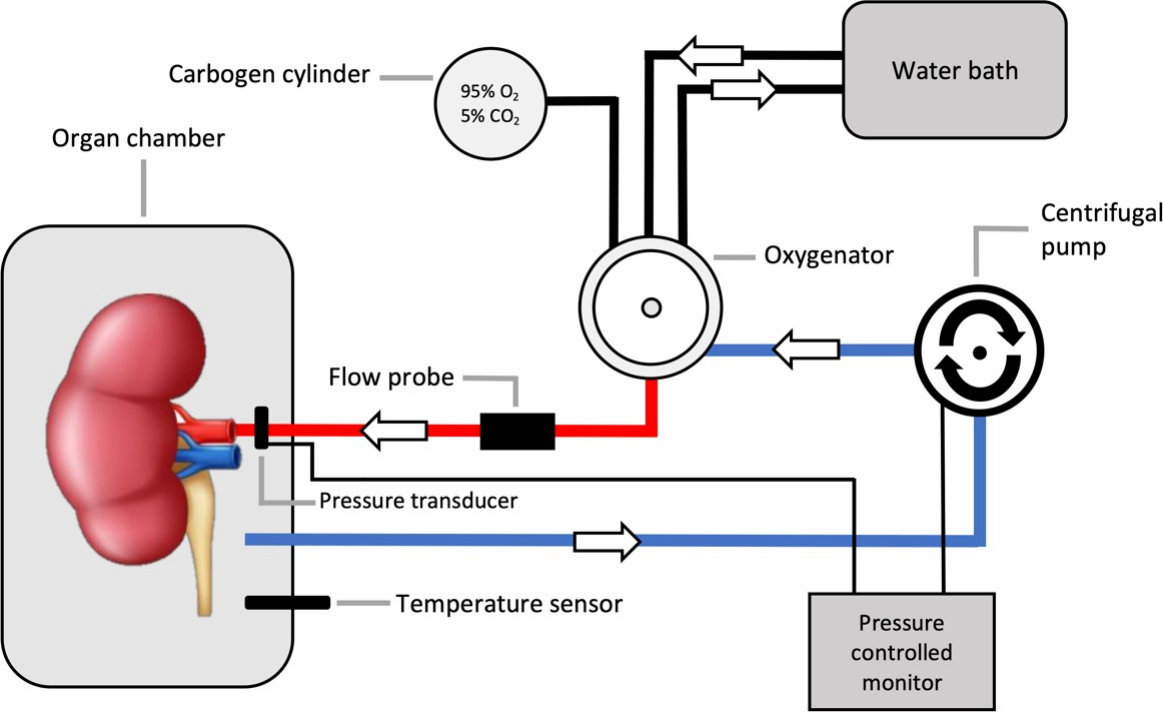
Schematic figure of the normothermic perfusion circuit.

The perfusion setup was identical to the one described previously by our group [14]. The kidneys were perfused in a sinusoid pulsatile fashion, with a frequency of 60 pulses per minute, at a set, non-feedback regulated, pressure of 110/70 mmHg and oxygenated with 95% oxygen/5% carbon dioxide (carbogen) during all experiments. This supraphysiological oxygen level is standard procedure in all four existing protocols. Perfusion was controlled by a custom-made electronic interface and control software (LabView Software, National Instruments Netherlands B.V., Woerden, the Netherlands). A schematic figure of the normothermic perfusion circuit is shown in Fig 1.

### Urine and perfusate analysis

Hourly, arterial perfusate and urine samples were taken. In groups 2 and 4, in which urine was recirculated, perfusate samples were taken prior to urine recirculation. Arterial blood gas samples of the perfusate were analysed after 0, 240 and 420 minutes of NMP. Perfusion parameters were documented every half hour. Concentrations of lactate dehydrogenase (LDH) and aspartate aminotransferase (ASAT), sodium, potassium, glucose and creatinine were measured with standard clinical assays in the perfusate. Creatinine clearance, fractional excretion of creatinine per 100g (FE creatinine/100g), and fractional sodium excretion (FENa^+^) were calculated to determine renal function. The equations can be found in the S1 Appendix. Both N-acetyl-β-D glucosaminidase (NAG) (also Sigma-Aldrich), as a marker of renal tubular dysfunction/injury, and thiobarbituric acid reactive substances (TBARS) (Lipid Peroxidation–malondialdehyde (MDA)) Assay Kit, Sigma-Aldrich B.V., Zwijndrecht, Netherlands), to quantify oxidative stress, were measured in the perfusate.

### Histology

A needle biopsy of the upper renal cortex was taken prior to the start of NMP. At the end of each experiment, surgical tissue samples from the upper cortex were collected. These biopsies were formalin-fixed, paraffin-embedded, cut, and stained with haematoxylin and eosin (HE) to assess changes in renal morphology as a result of NMP. A scoring method, based on existing histological scoring systems was developed [15], verified, and scored by an experienced clinical pathologist. All biopsies were scored on a scale of 0–3 on glomerular dilatation and tubular dilatation. A score of 0 indicated no signs of dilatation and a score of 3 indicated severe dilatation. A more elaborate analysis of renal tubular damage and acute tubular necrosis (ATN) was performed by two clinical pathologists, scoring necrosis on a scale ranging between 0–4 (0 = no sign, 1 = sporadic, 2 = cluster, 3 = confluent area’s, 4 = massive) with an additional classification of the morphology as severe and less severe (scaled as 0.67 and 0.33, respectively). Pro- and anti-apoptotic (Bax/Bcl2 ratio) gene expression levels were obtained from RNA-isolated snap-frozen biopsies using qPCR.

### Statistical analysis

Data analysis was performed using GraphPad Prism Version 8.3.1 (GraphPad software Inc., La Jolla, CA, USA). For all continuous longitudinally measured variables, such as ASAT and LDH, the area under the curve (AUC) was calculated. A one-way ANOVA with multiple comparisons was used to compare AUC values between groups if the data were normally distributed (Shapiro Wilk test) and had homogeneity of variances (tested by means of a Bartlett test). If data failed these assumptions the Kruskal-Wallis test with Dunn’s multiple comparisons test was used. Two-sided p-values of 0.05 or less were considered to indicate statistical significance.

## Results

### Perfusion parameters

In Table 2, data (mean, minimum and maximum) on warm and cold ischaemia, HMP duration and weight prior to the start and after NMP as well as delta weight (difference in weight at t = 420 versus t = 0) are presented. Delta weight was significantly higher in group 1 in comparison with group 2 (p = 0.041). There were no other significant differences between the values of all experimental groups.

**Table 2:** Baseline information.

	Group 1	Group 2	Group 3	Group 4
**WI (min)**	20 (20–20)	20 (20–20)	20 (20–20)	22 (20–24)
**CI (min)**	29.6 (19–46)	30.2 (21–49)	25.4 (20–38)	22 (16–32)
**HMP (min)**	142.4 (108–194)	143 (120–187)	161.1 (150–178)	145.6 (112–181)
**Weight at baseline (g)**	293 (267.8–324.7)	278.6 (230.4–324.3)	258.8 (221.9–282.3)	290.4 (253.9–349.6)
**Weight after NMP (g)**	464.8 (350–644.1)	344.4 (249.2–396.3)	401.7 (362–501.8)	424.2 (359.3–466.7)
**Delta weight (g)**	171.8 (82.2–319.4) 58% (31–98%)	65.75 (18.8–94.0) 23% (8–38%)	143 (79.7–222) 56% (28–79%)	133.8 (86.2–167.6) 47% (32–66%)

Mean (and minimum–maximum) warm ischaemia (WI) and cold ischaemia (CI) time, hypothermic machine perfusion (HMP) duration and weight of the kidneys in the four experimental groups.

**Fig 2:**
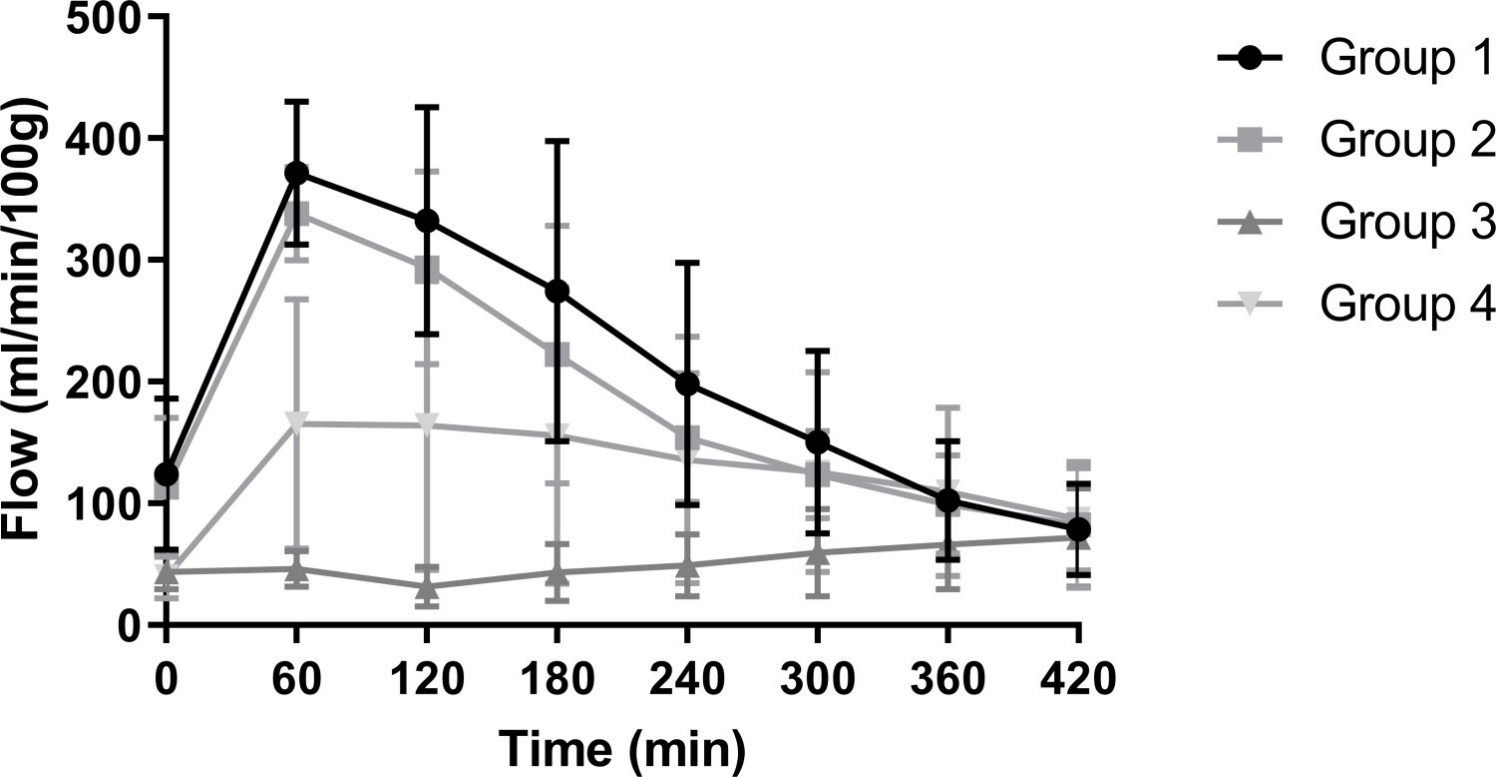
Renal arterial flow (ml/min/100g) of the four experimental groups during NMP (mean + SD).

During NMP renal arterial flow increased during the first 60 minutes of reperfusion in group 1 and 2. After 60 minutes flow rates typically started to decrease. Perfusions in group 3 and 4 started with lower flow values but showed a more constant flow throughout the NMP. End values after 7 hours of NMP were approximately 75 ml/min/100g in all groups (Fig 2).

### Urine and perfusate analysis

Individual data on functional and injury parameters throughout perfusion can be found in Table 3.

**Table 3:** Functional and injury parameters throughout perfusion.

Group	Time (min)	Serum creatinine (umol/l)	Creatinine clearance (ml/min)	FENa (%)	Albuminuria (g/l)	Absolute albuminuria (g)	LDH (mmol/l)	ASAT (mmol/l)	NAG (U/l)	MDA (μM)
**G 1 –Kidney 1**	60	390	0.66	24	<0.003	-	247	42	*	6.79
	180	377	1.25	3	<0.003	-	469	98	6.23	7.25
	300	350	1.10	41	<0.003	-	515	165	12.77	8.36
	420	375	0.40	95	<0.003	-	631	211	17.77	8.70
**G 1 –Kidney 2**	60	395	3.23	17	<0.003	-	246	57	1.93	5.96
	180	245	2.81	10	<0.003	-	420	79	3.03	6.17
	300	215	1.51	36	<0.003	-	535	99	4.85	6.18
	420	181	2.91	40	<0.003	-	510	128	8.80	5.93
**G 1 –Kidney 3**	60	474	1.05	92	<0.003	-	223	51	1.71	5.27
	180	416	1.38	45	<0.003	-	348	134	4.88	5.16
	300	382	0.58	91	<0.003	-	437	236	9.68	5.34
	420	353	0.37	90	<0.003	-	501	380	16.88	5.84
**G 1 –Kidney 4**	60	402	1.57	25	<0.003	-	567	43	2.22	4.18
	180	227	4.30	2	<0.003	-	642	61	2.99	5.00
	300	175	1.71	28	<0.003	-	666	71	5.01	4.86
	420	172	2.03	41	<0.003	-	618	73	10.24	6.49
**G 1 –Kidney 5**	60	372	1.41	52	<0.003	-	265	69	0.90	5.21
	180	333	0.72	25	<0.003	-	459	111	3.09	3.56
	300	250	1.95	61	<0.003	-	531	129	5.82	4.31
	420	248	2.12	124	<0.003	-	595	166	13.98	5.55
**G 2 –Kidney 1**	60	460	0.40	39	7.96	0.087	288	55	3.05	1.14
	180	455	0.15	4	8.79	0.009	498	95	4.26	1.44
	300	476	0.20	45	0.45	0.003	622	134	5.82	2.49
	420	497	0.05	59	0.86	0.002	706	175	9.02	2.59
**G 2 –Kidney 2**	60	646	0.06	82	1.14	0.004	332	59	1.77	1.15
	180	468	-	-	-	-	498	77	1.36	2.09
	300	500	-	-	-	-	619	95	2.39	2.76
	420	542	2.60	7	0.12	0.001	770	125	7.27	5.12
**G 2 –Kidney 3**	60	441	0.76	17	8.93	0.089	232	40	2.56	1.34
	180	433	-	-	-	-	421	72	2.24	1.69
	300	572	-	-	-	-	687	111	3.15	2.34
	420	508	-	-	-	-	733	113	6.59	2.01
**G 2 –Kidney 4**	60	358	5.53	6	8.09	0.267	208	41	2.48	0.87
	180	313	1.91	3	4.93	0.039	447	136	2.70	1.92
	300	369	0.18	85	0.24	0.002	486	134	3.41	5.27
	420	413	0.34	98	0.23	0.004	607	161	6.73	7.41
**G 2 –Kidney 5**	60	607	-	-	-	-	291	36	2.18	2.44
	180	558	-	-	-	-	337	45	1.64	2.52
	300	603	-	-	-	-	410	63	1.44	5.49
	420	603	-	-	-	-	453	78	2.61	5.55
**G 3 –Kidney 1**	60	1241	1.18	263*	0.02	0.004	362	101	6.03	1.64
	180	764	0.14	56	0.01	0.000	559	161	18.25	1.52
	300	567	4.07	35	0.02	0.003	907	285	33.17	<0.5
	420	372	3.50	45	0.01	0.001	875	356	47.53	<0.5
**G 3 –Kidney 2**	60	788	4.00	78	0.04	0.005	429	95	5.27	2.67
	180	708	1.41	77	0.04	0.002	804	202	15.01	1.86
	300	547	2.29	82	0.04	0.005	951	334	21.73	1.78
	420	311	3.22	81	0.04	0.006	906	651	42.06	1.70
**G 3 –Kidney 3**	60	1254	0.28	80	0.04	0.000	333	70	2.59	2.36
	180	820	1.71	75	0.04	0.002	432	87	5.80	2.40
	300	803	2.08	80	0.04	0.004	613	207	11.02	2.42
	420	564	1.77	99	0.04	0.004	785	638	33.15	1.97
**G 3 –Kidney 4**	60	912	4.80	67	0.02	0.004	434	77	3.83	1.42
	180	554	3.38	56	0.01	0.001	704	148	9.11	1.13
	300	474	1.76	75	0.03	0.003	940	449	15.80	1.94
	420	275	2.36	87	0.03	0.004	1018	856	27.32	1.37
**G 3 –Kidney 5**	60	628	10.77	49	0.04	0.011	233	39	3.08	0.90
	180	324	6.30	77	0.04	0.010	447	95	8.25	0.79
	300	119	8.40	63	0.04	0.012	305	222	6.54	<0.5
	420	125	2.67	117	0.02	0.004	725	796	13.12	0.69
**G 4 –Kidney 1**	60	711	1.76	47	4.50	0.225	384	62	2.97	2.36
	180	603	3.68	26	4.20	0.399	818	339	10.44	2.89
	300	593	3.47	29	6.11	0.580	1247	882	22.77	6.05
	420	564	3.25	39	10.17	1.017	1363	937	32.53	7.98
**G 4 –Kidney 2**	60	423	0.99	38	12.76	0.319	452	135	1.56	3.73
	180	401	2.54	19	5.02	0.236	882	377	14.19	4.87
	300	420	1.04	30	7.93	0.230	1119	833	25.87	9.89
	420	395	1.52	16	12.03	0.271	1302	983	27.85	5.94
**G 4 –Kidney 3**	60	598	1.84	32	18.11	0.598	290	58	1.64	2.01
	180	488	3.57	12	1.67	0.100	623	153	4.21	1.98
	300	433	4.04	11	2.22	0.111	1064	228	7.70	6.13
	420	432	3.82	18	3.14	0.207	1220	264	11.79	9.80
**G 4 –Kidney 4**	60	491	5.43	23	10.04	0.803	241	51	1.69	3.08
	180	416	4.67	9	3.76	0.199	558	176	5.69	3.60
	300	402	5.12	11	6.75	0.439	797	263	8.29	4.37
	420	410	3.66	20	9.15	0.549	855	313	9.52	7.80
**G 4 –Kidney 5**	60	457	0.27	31	10.87	0.054	279	57	1.57	4.28
	180	470	0.26	43	13.07	0.105	473	114	4.31	2.87
	300	457	0.29	49	18.81	0.188	653	262	10.08	13.31
	420	437	0.19	61	19.56	0.137	808	493	21.59	11.86

#### Renal function

**Fig 3:**
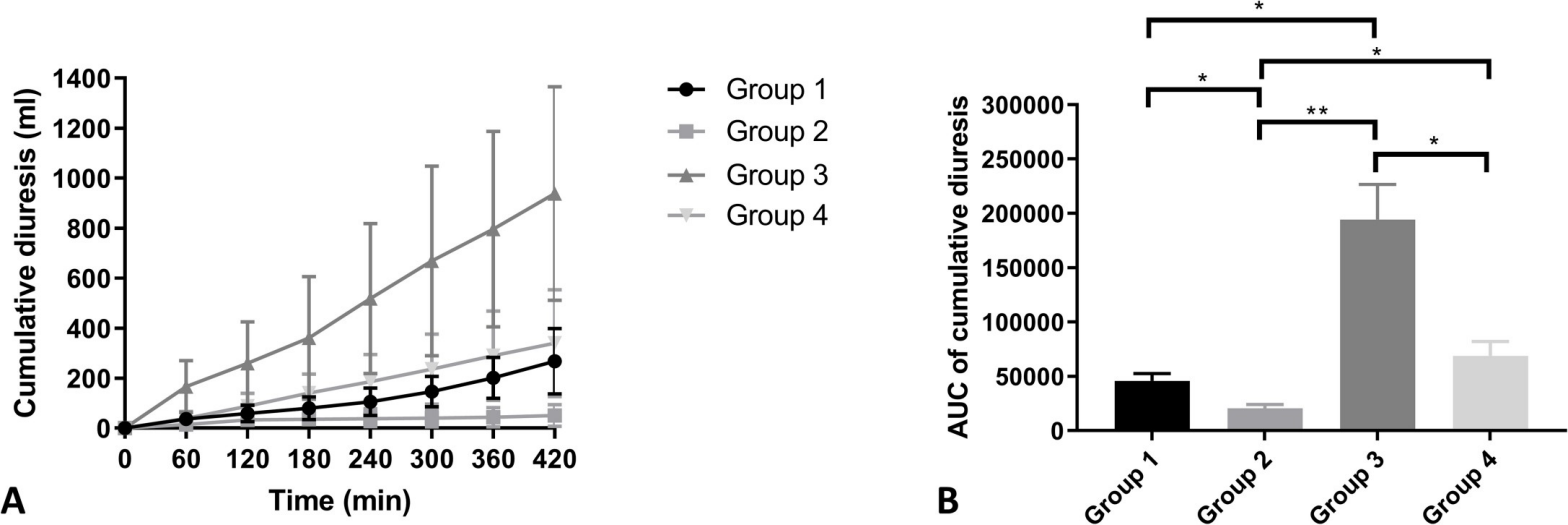
Cumulative diuresis (ml) (A) and AUC (B) of the four experimental groups during NMP (mean + SD). ** indicates a p-value of ≤ 0.01. *** indicates a p-value of ≤ 0.001.

Group 3 showed the highest levels of urine output (Fig 3). Cumulative diuresis in group 3 was significantly higher in comparison with group 1 (p = 0.003), group 2 (p < 0.001) and group 4 (p = 0.008). Some of the kidneys in group 2 did not produce urine at t = 180 and t = 300, leading to lower average levels of cumulative urine production at t = 360 and t = 420.

**Fig 4:**
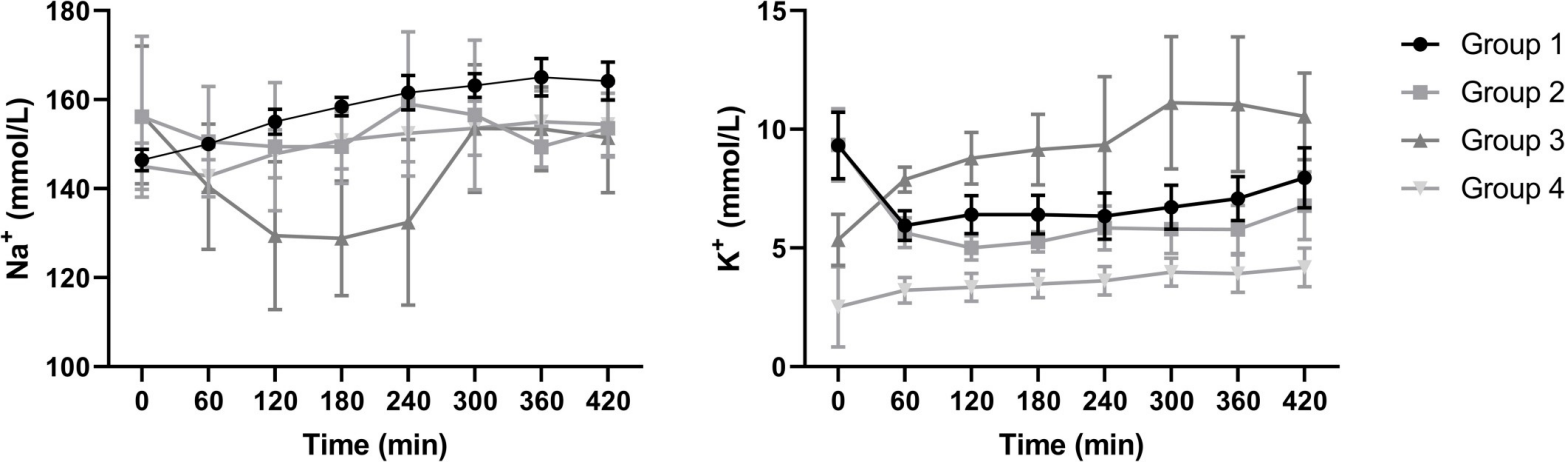
Sodium and potassium levels (mmol/L) of the four experimental groups during NMP (mean + SD).

Sodium and potassium levels differed considerably between groups (Fig 4). The levels at t = 0 indicate the initial starting values at perfusion. Especially group 3 showed highly fluctuating sodium and potassium levels throughout perfusion, whereas levels in the other groups remained more stable.

**Fig 5:**
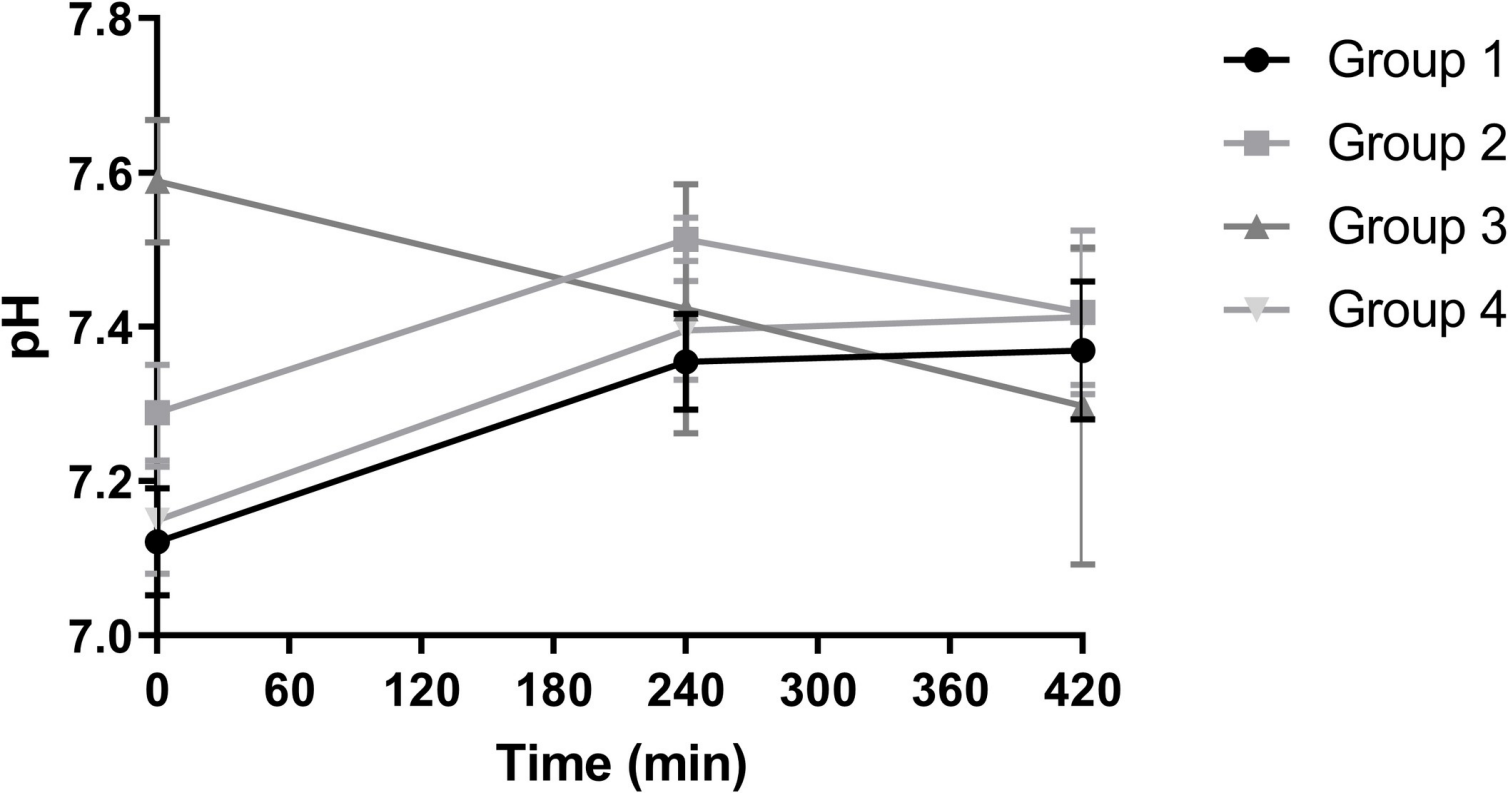
pH of the four experimental groups during NMP (mean + SD).

During perfusion, pH in group 3 declined without stabilisation whereas the other groups reached a more balanced level of approximately 7.4 after 7 hours of NMP (Fig 5).

**Fig 6:**
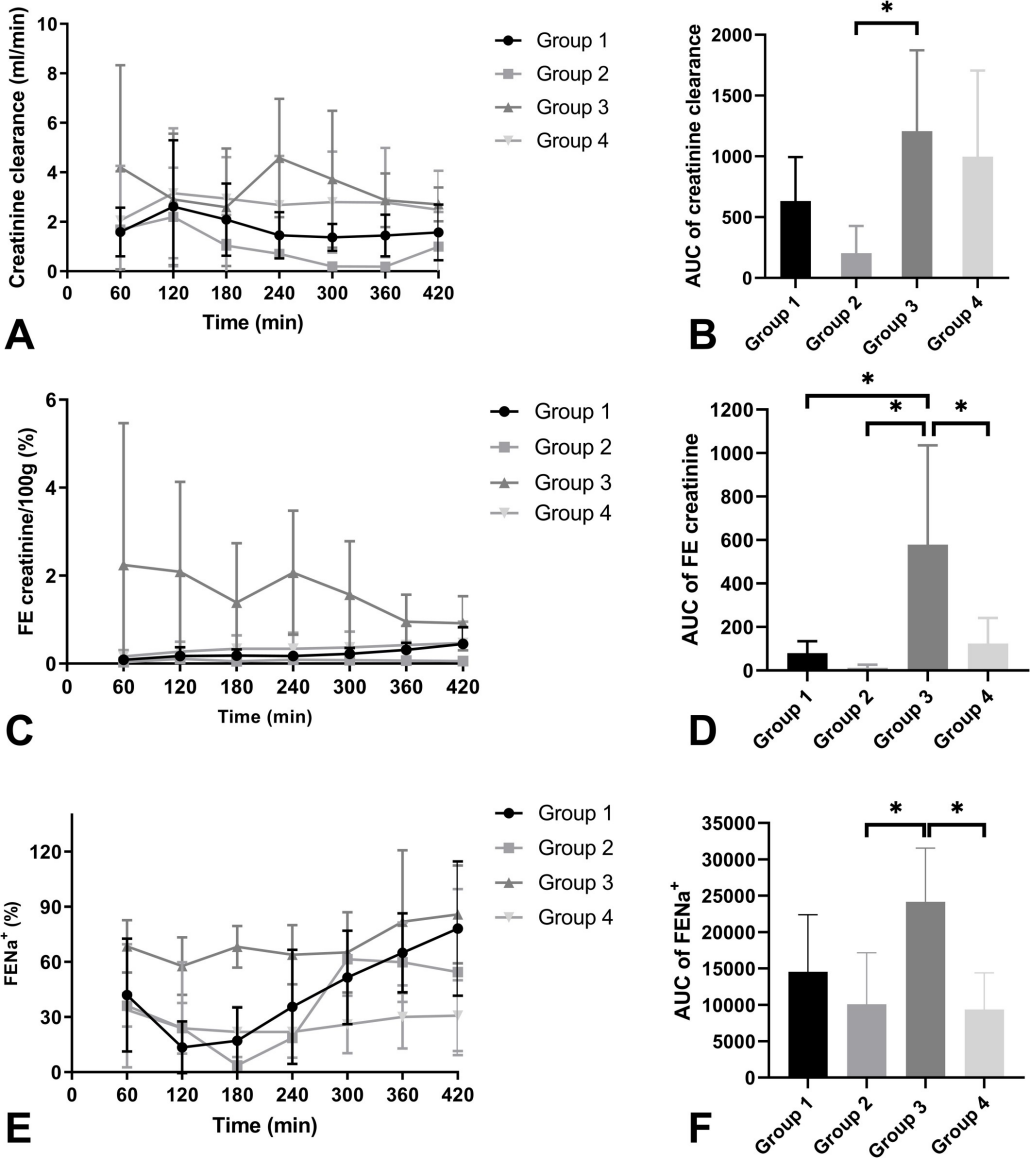
Mean creatinine clearance (ml/min) (A), AUC of creatinine clearance (B), mean fractional excretion of creatinine per 100g (FE creatinine/100g) (C), AUC of FE creatinine (D), mean fractional excretion of sodium (FENa^+^) (E), and AUC of FENa^+^ (F) of the four experimental groups during NMP (+ SD). * indicates a level of significance of p ≤ 0.05.

Creatinine clearance was low in all groups (Fig 6A and 6B). Nevertheless, it was significantly higher than in group 3 compared to group 2 (p = 0.039). Fractional excretion of creatinine per 100g (Fig 6C and 6D) was significantly higher in group 3 compared to group 1 (p = 0.026), group 2 (p = 0.017), and group 4 (p = 0.045). FENa^+^ was high in all groups, indicating that tubular function was severely impaired (Fig 6E and 6F) in these ischaemically damaged kidneys. FENa^+^ in group 3 was significantly higher compared to group 2 (p = 0.037) and group 4 (p = 0.019). All other groups did not differ significantly.

#### Renal injury

**Fig 7:**
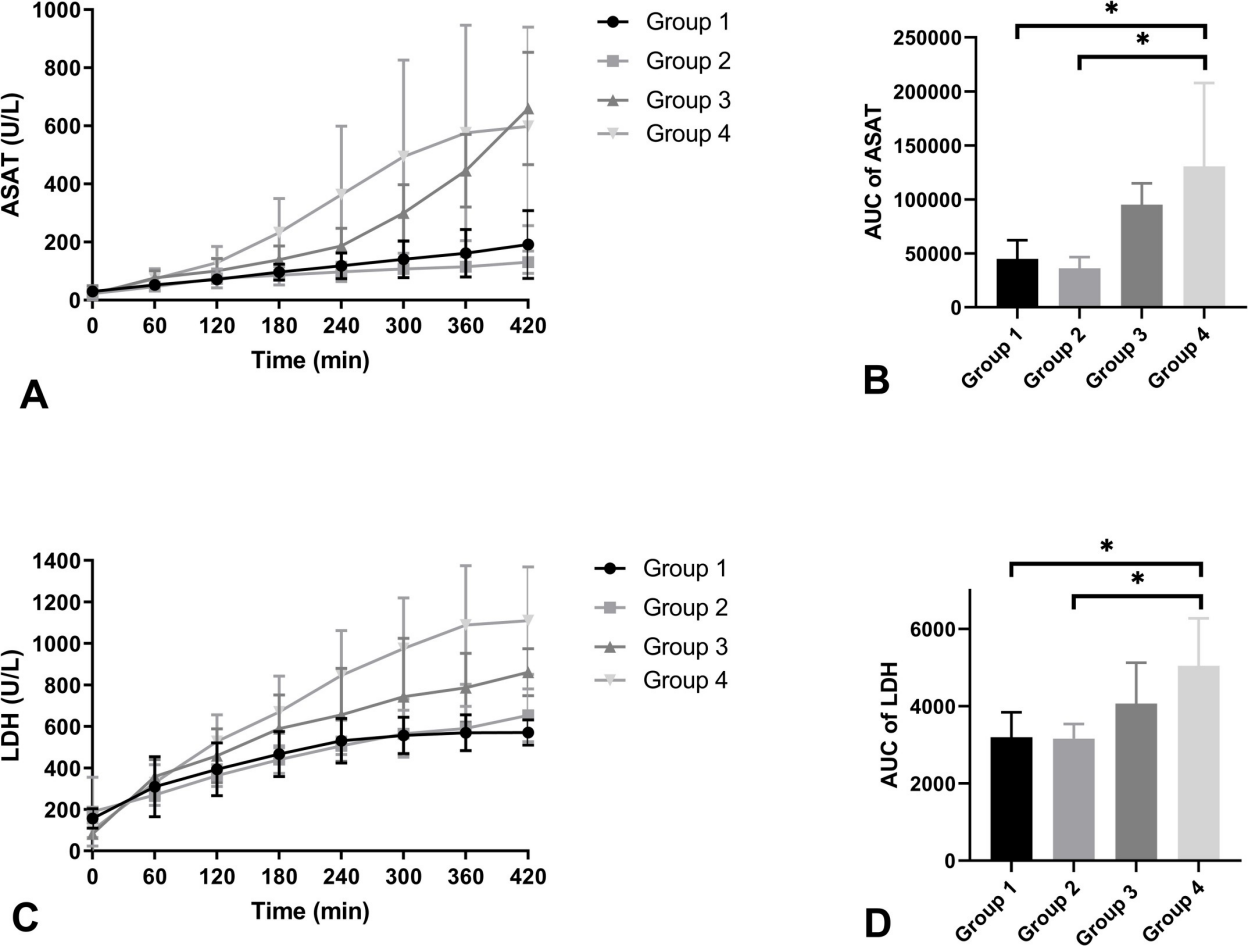
ASAT levels (U/l) (A), AUC of ASAT (B), LDH levels (U/l) (C), and AUC of LDH (D) of the four experimental groups during NMP (mean + SD). * indicates a level of significance of p ≤ 0.05.

ASAT levels during NMP were analysed (Fig 7A and 7B) as a marker for general cell injury. The mean AUC was determined for each group. Levels in group 4 were significantly higher compared to group 1 (p = 0.022) and group 2 (p = 0.011). LDH levels were also measured in the perfusate as a general injury marker (Fig 7C and 7D). An AUC was calculated which showed lowest levels of LDH in groups 1 and 2. Group 2 was significantly lower compared to group 4 (p = 0.020). Group 1 showed significantly lower levels compared to group 4 (p = 0.022).

**Fig 8:**
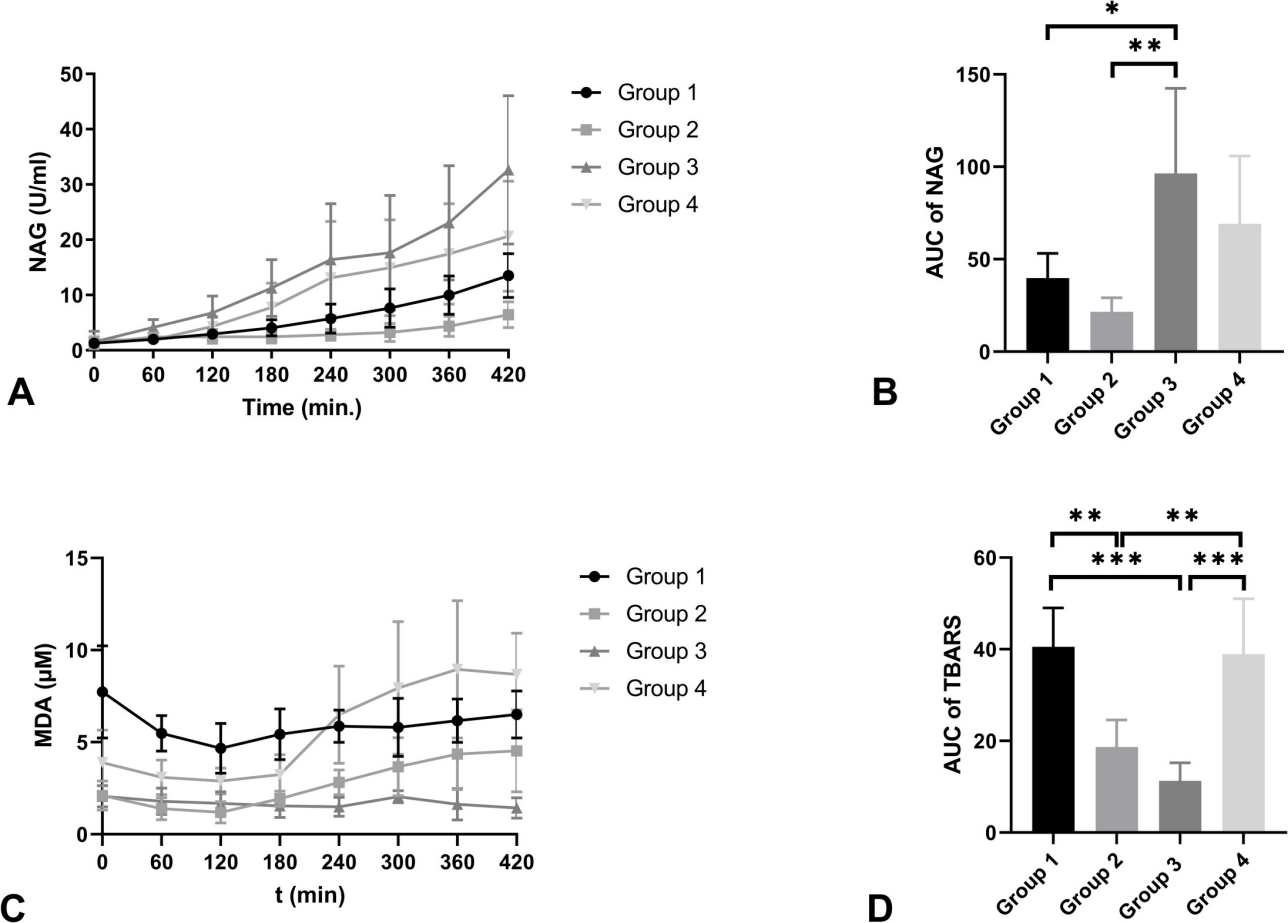
Mean NAG levels (U/L) (A) and AUC of NAG (B), mean MDA levels (uM) (C), and AUC of MDA (D) of the four experimental groups during NMP (+ SD). * indicates a level of significance of p ≤ 0.05. ** indicates a p-value of ≤ 0.01. *** indicates a p-value of p ≤ 0.001.

NAG levels were significantly higher in group 3 in comparison with group 1 (p = 0.043) and group 2 (p = 0.006) (Fig 8A and 8B). TBARS, the concentration of malondialdehyde (MDA), were measured in all four groups as a marker for lipid peroxidation (Fig 8C and 8D). MDA levels were significantly higher in group 1 when compared to group 2 (p = 0.003) and group 3 (p < 0.001). Group 2 showed significantly lower MDA levels compared to group 4 (p = 0.006). Group 3 showed significantly lower levels compared to group 4 (p <0.001).

### Histology

**Fig 9:**
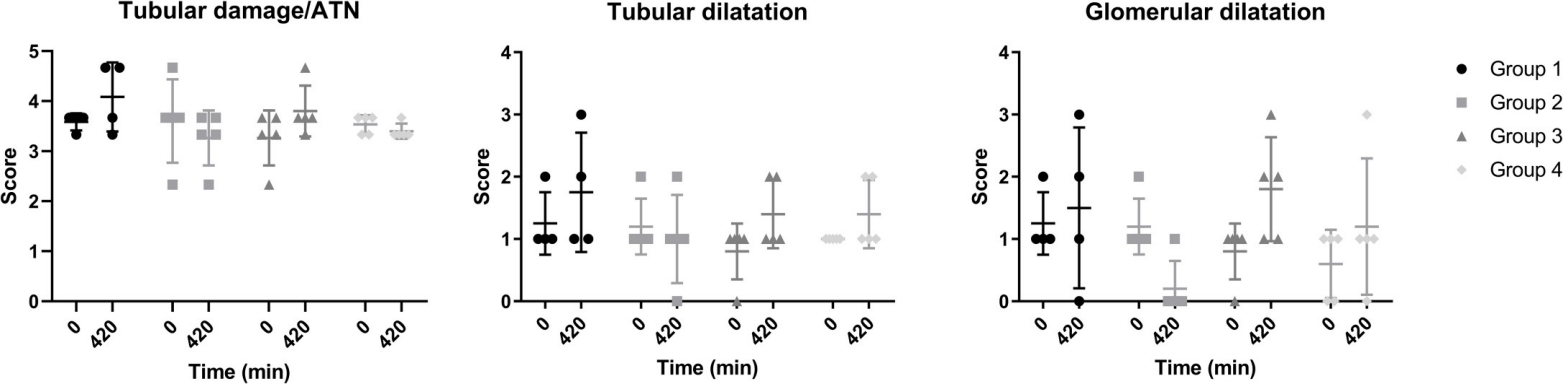
Scores on necrosis, tubular and glomerular dilatation at t = 0 and t = 420 (mean + SD).

The dot plots in Fig 9 show renal tubular damage/ATN, tubular dilatation, and glomerular dilatation scores before the start of NMP (t = 0) and after 7 hours of NMP (t = 420). Initial values of histological necrosis and tubular dilatation were comparable between groups, with only marginal differences in end-point scores. Glomerular dilatation at t = 420 was remarkably lower in group 2 in comparison with the other 3 groups. Histological figures per group can be found in the S1 Fig.

**Fig 10:**
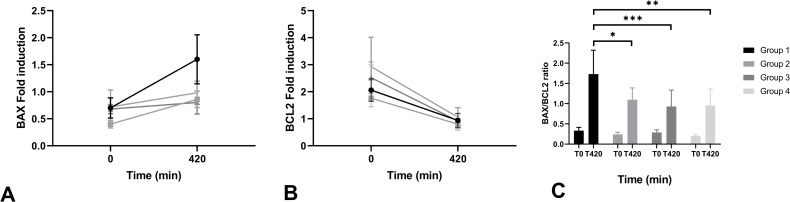
Bax (A) and Bcl-2 (B) levels at t = 0 and t = 420 (mean + SD). Bax/Bcl-2 ratios (C) are displayed per group. * indicates a level of significance of p ≤ 0.05. ** indicates a p value of ≤ 0.01. *** indicates a p value of p ≤ 0.001.

Bax and Bcl-2 fold induction, indicators for pro- and anti-apoptotic gene expression, respectively, both showed a shift in end-point NMP levels compared to levels at t = 0 (Fig 10). Delta Bax gene expression (difference between t = 0 and t = 420) was calculated in all groups and was significantly higher in group 1 compared to group 2 (p = 0.046), group 3 (p < 0.001) and group 4 (p = 0.003). Bcl-2 expression decreased in all groups after 7 hours of perfusion with no significant differences between groups.

## Discussion

General interest in renal normothermic ex-vivo machine perfusion techniques is rising, but clinical evidence remains limited with only one ongoing clinical NMP trial. Extensive pre-clinical studies have been conducted investigating the potential of NMP. A plethora of different renal normothermic perfusates exists among different research groups. However, the question to what extent the perfusate composition impacts renal function and how it affects the interpretation of potential viability markers remains. Therefore, this study evaluated the influence of four different normothermic ex-vivo perfusion solutions side-by-side on renal function and kidney injury during prolonged NMP using a porcine kidney donation after circulatory death model.

To date, decision-making strategies to predict post-transplant renal function lack overall predictive power and contain an element of subjectivity. Normothermic perfusion could potentially serve as an add-on strategy to improve and objectify current clinical decision-making methods. The restoration of cellular metabolism at normothermia entails the possibility to assess isolated renal function prior to transplantation. Hosgood and colleagues provided the first clinical evidence of the feasibility of pre-transplant viability assessment during renal NMP. Their perfusion solution is currently the most widely used during NMP and was initially developed to assess renal function prior to transplantation within a 1–2 hour timespan [16]. Kidneys perfused with this solution usually show high urine production [17]. Indeed, the highest urine production was observed in group 3, which is based on this clinical UK perfusate, leading to a more than complete urinary excretion of the circulating volume of the perfusate in this group during 7 hours of NMP. The continuous loss of circulating perfusion fluid volume had to be compensated by intermittent addition of similar volumes of Ringer’s lactate, conforming to the clinical protocol for this perfusion solution. In our study, this resulted in considerable alterations of the electrolyte composition and pH, since urine was not recirculated. This high urinary production can most likely be attributed to the absence of a colloid in only this perfusion solution. All three other groups’ perfusion solutions contained a considerable amount of albumin. In the intravascular space, albumin is the main component that maintains a normal colloid osmotic pressure [18]. The balance between hydrostatic pressure and colloid osmotic pressure results in a physiological ultrafiltration rate over the glomerular membrane [19]. Kaths et al. showed that urine production during NMP did not correlate with post-transplant function. They hypothesised that urine production is largely influenced by the perfusate composition, in particular the oncotic pressure and osmolarity, which is in line with our results [20].

The main responsible organ for the regulation of electrolytes is the kidney but there is evidence that the kidney itself also benefits from a balance in electrolytes. It has been established that an imbalance in potassium and specifically hypokalaemia, can lead to a variety of changes in renal function including impaired tubular transport, impaired urinary concentrating ability, altered sodium reabsorption, and intracellular acidosis [21–23]. To account for the inevitable urinary loss of important perfusate electrolytes, Weissenbacher et al. proposed the use of urine recirculation during NMP. They showed that it facilitates proper maintenance of perfusate volume and homeostasis in discarded human kidneys, even after 24 hours of NMP [7].

The measured albumin concentration in the perfusate decreased over time during perfusion. This decrease is most likely caused by the adherence of albumin to the plastic perfusion circuit tubing and only a small amount can be attributed to the loss in the produced urine, which is also observed in other NMP studies [24]. This urinary loss of albumin may be explained by warm ischaemia-induced damage to endothelial cells with a disruption of the filter diaphragms resulting in proteinuria, which is in line with our observed levels of albuminuria during NMP [25].

We cannot fully explain the mechanism of the differences between groups that we observed in kidney weight gain during NMP. Histological examination was not decisive on possible areas of oedema formation. Further investigation is needed to clarify the potentially multifactorial mechanisms of weight gain in relation to the perfusion solution used.

Various perfusion parameters were measured, where renal flow showed the most striking differences. Renal flow in groups 3 and 4 maintained the most stable levels throughout the 7-hour NMP period. The solutions in these groups were supplemented with a vasodilator while solutions in the first two groups were not. Endogenous vasodilators promote vascular smooth muscle relaxation and thereby regulate regional blood flow [26]. As groups 1 and 2 were not supplemented with a vasodilator, vasospasms could have resulted in reduced renal arterial flow. Moreover, there is evidence that medullary and cortical flow can be regulated individually [27,28]. Preliminary unpublished data from our group, based on precise and quantitative functional MRI (ASL) flow measurements during NMP strongly supports this mechanism. In those experiments, renal flow during the first hour of NMP is mainly medullar, which shifts to cortical flow after more than an hour of perfusion. This regional redistribution of flow might explain the peak seen in perfusions of groups 1 and 2, possibly due to medullary shunting during the first hour. The addition of a vasodilator at the start of NMP could enhance a predominantly cortical microperfusion immediately at the start of NMP, resulting in a more stable flow as observed in group 3 and 4. Hence, if a stable flow pattern and a possibly more physiological predominantly cortical perfusion are desired during NMP, it may be advisable to add a vasodilator to the perfusion solution. Alternatively, repeated washing of RBCs prior to perfusion could reduce renal vasoconstriction by diminishing the vasoconstrictive activity of RBCs [29].

Kidney function during NMP was markedly impaired in all porcine kidneys, possibly due to the initial warm ischaemic damage and the lack of physiological humoral regulatory mechanisms, such as those driven by anti-diuretic hormone and aldosterone. Creatinine clearance was low and high values of FENa^+^ were observed in all groups. The sudden increase in FENa^+^ after 3 hours of NMP in group 2 could have been the result of the absence of urine production in several kidneys at t = 240 and t = 300, while at the end of perfusion kidneys started to produce urine again. Elevated values of FENa^+^ is presumably the result of tubular necrosis as has also been reported in previous studies [30,31]. In addition, in-vivo creatinine clearance and FENa^+^ levels are influenced to a great extent by humoral regulation. As humoral regulation lacked during the NMP experiments, physiological creatinine clearance and FENa^+^ levels are not to be expected in our NMP setup.

To quantify injury to renal cells, ASAT and LDH levels were measured. The highest levels were observed in groups 3 and 4, suggesting that most injury occurred in these two groups. Kaths et al. showed that increased ASAT levels during NMP correlate with post-transplant renal function and therefore ASAT might be an important organ viability assessment biomarker [20]. The influence of different perfusate compositions on measured ASAT levels emphasises the importance to harmonise NMP protocols among transplant centres, to enable consistent interpretation of NMP data on a global basis. Levels of NAG, a marker for altered tubular integrity and tubular damage [32,33], were highest in group 3 indicating that most tubular injury occurred in this group. TBARS levels were measured to quantify oxidative stress during perfusion. The highest levels were observed in group 1 and 4, suggesting that kidneys in these groups experienced the highest oxidative stress, which could have resulted in impaired cellular function [34]. The shift in Bax/Bcl-2 ratio after 7 hours of NMP compared to the biopsy prior to NMP is probably the result of the initial warm ischaemic period, rather than the potentially damaging effects occurring during NMP. Ischaemic injury following consequent reperfusion induces a regulatory apoptosis cascade, which plays a key role in the process of repair of the damaged proximal tubule [35]. Although many normothermic injury markers have been proposed, to date none have been validated in a well-powered clinical trial. A study correlating an array of promising injury markers measured during NMP with post-transplant renal function is required to elucidate which potential biomarkers should be incorporated in normothermic ex-vivo viability assessment criteria.

Our NMP setup was used in conjunction with a porcine model of DCD kidney donation in which slaughterhouse kidneys were utilised. A limitation of this study is that a slaughterhouse procedure is based on exsanguination followed by cardiac arrest, which makes it slightly different from an actual DCD model in which circulatory arrest results from cardiac ischaemia and/or failure. However, we observed that kidneys in our study showed ischaemic injury similar to that incurred after DCD [36,37]. Hence, we feel that the present slaughterhouse DCD model will help reduce the use of laboratory animals while maintaining essential reliability and reproducibility. Although our experimental groups were relatively small, our group sizes are comparable to those in other porcine kidney machine perfusion studies [17,38–40].

The NMP perfusion parameters that we recorded in group 3, which was based on the British clinical perfusion solution used by Hosgood et al., cannot be fully compared to results previously reported by the Leicester/Cambridge group [6,12]. In our study, kidneys underwent a much longer period of NMP and were perfused with a higher mean arterial pressure. Furthermore, kidneys perfused by Hosgood et al. were transplanted after machine perfusion. For a fully reliable comparison between the four perfusion solutions, a future study should include transplantation of kidneys after NMP as this will allow for actual follow-up and functional assessment in vivo.

In conclusion, perfusion of porcine kidneys proved feasible with all four solutions tested. However, there were substantial differences between electrolyte levels, renal function parameters, and injury markers in the four groups. These differences, in combination with current heterogeneity in applied perfusion solutions among groups, impedes the developments of standardised NMP assessment criteria. We feel that in both experimental and clinical NMP it is essential to carefully pre-specify the exact purpose and desired duration of NMP, as each could call for necessary adjustments in the perfusate and perfusion protocol. It is important to emphasise that this study focussed on the effects of existing perfusates *as a whole* on measured outcomes, rather than investigating the roles of individual perfusate components. Further studies are required to elucidate the individual influences and merits of each perfusate ingredient during NMP.

## Supporting information

S1 Fig Histology 4 perfusate groups. (PDF)

S1 Appendix Renal function equations. (DOCX)

S1 Data (PDF)
