# Supplementary material for: Prolonged ex-vivo normothermic kidney perfusion: The impact of perfusate composition
Source: PLoS One. 2021 May 18;16(5):e0251595. doi: 10.1371/journal.pone.0251595 (PMC8130974; doi:10.1371/journal.pone.0251595)
Supplement: S1 Fig — (PDF) [file pone.0251595.s001.pdf]

S1 Fig. Histology 4 perfusate groups

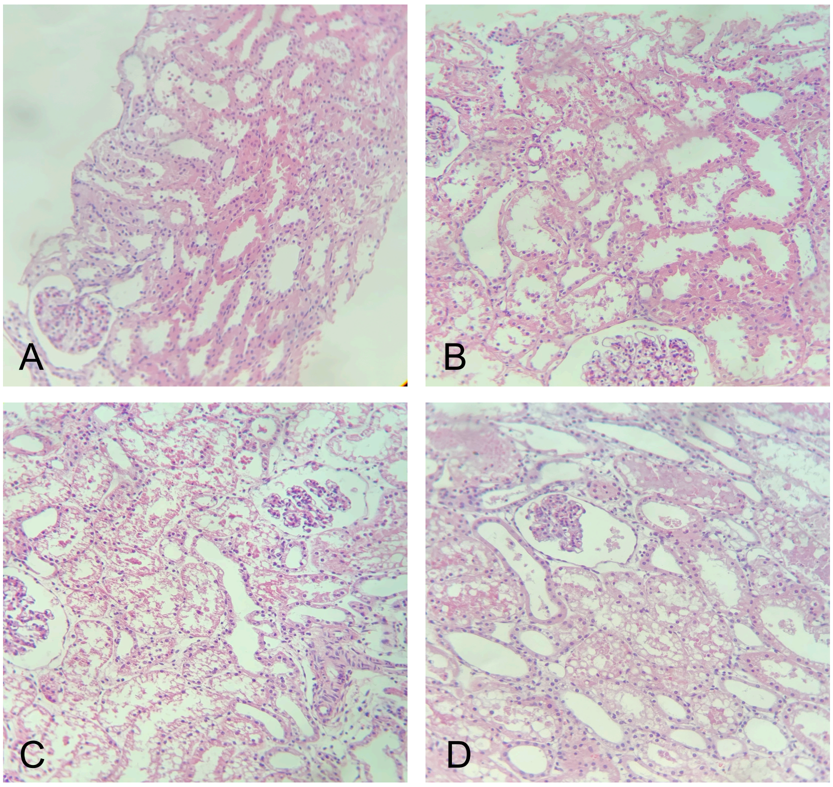

Fig 1. Histology (HE stained) group 1. t = 0 biopsy (A&B) and t = 420 (C&D).

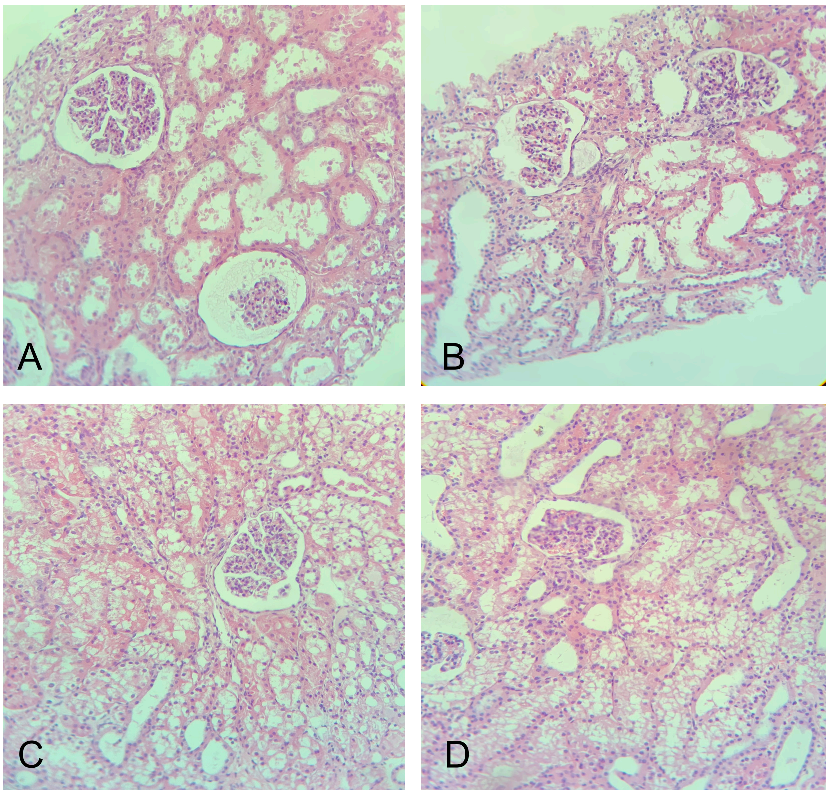

Fig 2. Histology (HE stained) group 2. t = 0 biopsy (A&B) and t = 420 (C&D).

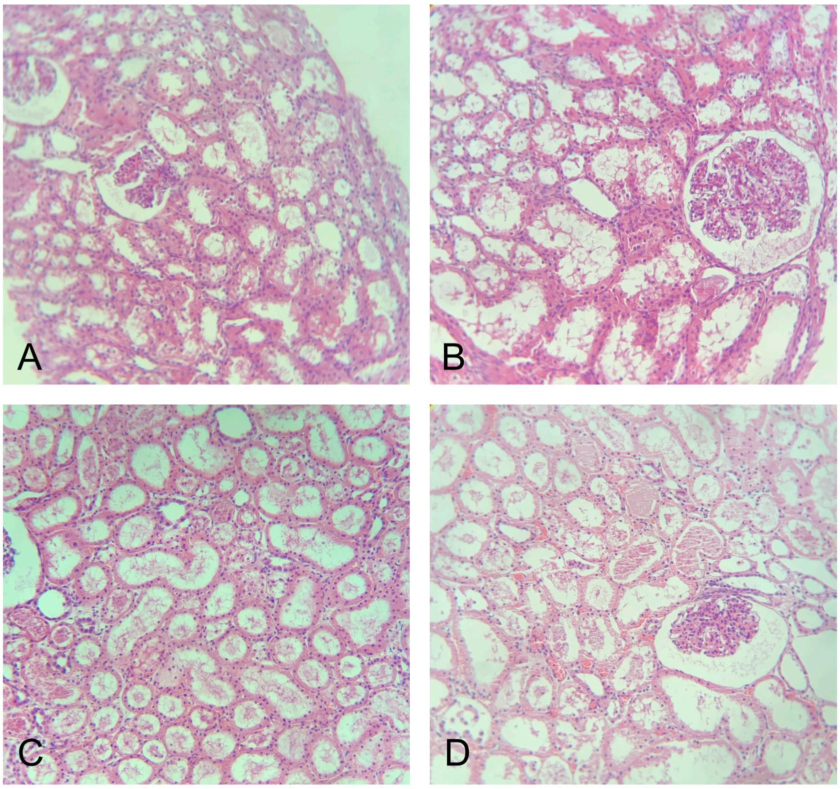

Fig 3. Histology (HE stained) group 3. t = 0 biopsy (A&B) and t = 420 (C&D).

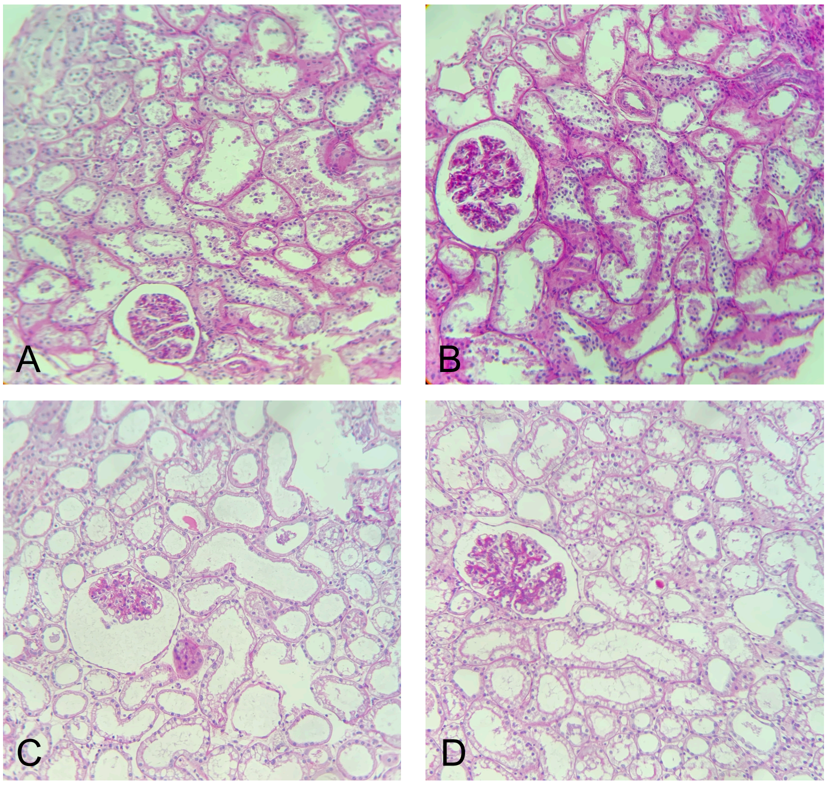

Fig 4. Histology (HE stained) group 4. T = 0 biopsy (A&B) and T = 420 (C&D).
