# Supplementary material for: Prolonged ex-vivo normothermic kidney perfusion: The impact of perfusate composition
Source: PLoS One. 2021 May 18;16(5):e0251595. doi: 10.1371/journal.pone.0251595 (PMC8130974; doi:10.1371/journal.pone.0251595)
Supplement: S1 Appendix — (DOCX) [file pone.0251595.s002.docx]

### **S1 Appendix – Renal function equations**

**Creatinine clearance (mL/min):**

$$\left( \frac{U_{creatinine}}{P_{creatinine}} \right) x diuresis$$

Urinary and perfusate creatinine concentration in mmol/L; diuresis in mL/min.

**Fractional excretion of creatinine (%/100g):**

$$FEcreatinine=\frac{{Creatinine}_{excretion}}{{Creatinine}_{delivery}}$$

$$\begin{aligned} FEcreatinine=\frac{\left( \frac{{Flow}_{urine} *{Creatinine}_{urine}}{{Flow}_{perfusate}*\left( 1-hematocrit \right)*{Creatinine}_{serum}} \right)}{renal weight}*{10}^{4} \end{aligned}$$

Urinary and perfusate flow in L/min; urinary and serum creatinine in umol/L; renal weight in grams.

**Fractional excretion of sodium (%):**

$$\left( \frac{U_{Na} x P_{creatinine}}{P_{Na}x U_{creatinine}} \right)x 100$$

Urinary and perfusate sodium concentration in mmol/L; perfusate and urinary creatinine concentration in µmol/L.
