# Supplementary material for: Prolonged ex-vivo normothermic kidney perfusion: The impact of perfusate composition
Source: PLoS One. 2021 May 18;16(5):e0251595. doi: 10.1371/journal.pone.0251595 (PMC8130974; doi:10.1371/journal.pone.0251595)
Supplement: S1 Data — (PDF) [file pone.0251595.s003.pdf]

			Bloodgas					Perfusate		
Experiment	Flow (ml/min)	t (min)	Na+ (mmol/L)	pH	pCO2 (kPa)	pO2 (kPa)	Lac (mmol/L)	AST (U/L)	creat (umol/L)	Na+ (mmol/L)
**WME**	554	0	145	7,05	6,62	72,30	6,7	33	394	149
Group 1	955	60						42	390	149
	670	120						64	391	154
	446	180						98	377	157
	330	240	156	7,282	6,11	71,80	5,6	137	373	164
	231	300						165	350	161
	125	360						183	358	163
	78	420	156	7,233	6,50	65,40	7,5	211	375	163
**WME**	380	0	140	7,112	7,20	>73,3	2,9	36	643	144
Group 1	1000	60						57	395	150
	800	120						67	281	158
	420	180						79	245	161
	250	240	159	7,384	7,09	57,40	1,5	83	233	166
	215	300						99	215	167
	185	360						104	198	169
	165	420	162	7,427	6,53	59,60	2,5	128	181	170
**WME**	153	0	136	7,19	6,08	>73,3	2,6	25	630	149
Group 1	1070	60						51	474	149
	994	120						86	436	152
	920	180						134	416	156
	655	240	152	7,294	7,40	65,20	1,6	174	388	158
	490	300						236	382	161
	290	360						285	359	159
	222	420	153	7,323	6,42	65,70	3,4	380	353	159
**WME**	523	0	145	7,198	6,75	>73,3	1,6	21	624	145
Group 1	1287	60						43	402	152
	1305	120						52	290	158
	1182	180						61	227	160
	916	240	160	7,402	6,95	62,60	2,9	64	184	163
	699	300						71	175	165
	422	360						73	178	169

	313	420	162	7,432	6,87	66,30	2,8	73	172	167
**WME**	200	0	140	7,06	9,61	>73,3	1,3	34	712	145
Group 1	1095	60						69	372	150
	1055	120						87	347	153
	1019	180						111	333	158
	727	240	152	7,413	6,28	>73,3	3,1	131	309	157
	545	300						129	250	162
	476	360						161	246	165
	370	420	153	7,434	6,36	>73,3	3,2	166	248	162
**Groningen**	217	0	125	7,237	7,02	88,90	2,1	30	696	137
Group 2	849	60						55	460	143
	790	120						77	395	139
	660	180						95	455	150
	513	240	140	7,498	5,64	52,70	2,5	107	452	147
	523	300						134	476	151
	451	360						152	489	150
	402	420	145	7,329	9,52	73,20	3,2	175	497	155
**Groningen**	560	0	132	7,285	7,16	59,30	2,2	20	904	156
Group 2	1013	60						59	646	172
	1070	120						65	450	140
	815	180						77	468	141
	490	240	138	7,479	6,23	61,50	2,6	86	498	144
	333	300						95	500	143
	205	360						106	530	145
	126	420	137	7,285	9,45	71,90	7,1	125	542	144
**Groningen**	165	0	137	7,306	7,01	87,10	1,7	14	1141	186
Group 2	990	60						40	441	142
	930	120						64	387	150
	403	180						72	433	148
	358	240	145	7,532	5,53	75,30	2,8	83	450	151
	361	300						111	572	186
	405	360						98	464	147
	444	420	148	7,521	5,72	65,10	4,3	113	508	162

**Groningen**	402	0	131	7,232	7,06	89,60	1,4	5	910	152
Group 2	930	60						41	358	151
	940	120						127	303	174
	922	180						136	313	163
	560	240	147,0	7,550	5,41	76,10	2,7	149	384	174
	315	300						134	369	151
	182	360						148	393	157
	110	420	149	7,505	5,96	75,40	3,5	161	413	155
**Groningen**	212	0	140	7,385	7,37	86,70	1,6	41	819	150
Group 2	879	60						36	607	145
	402	120						40	568	144
	270	180						45	558	145
	217	240	144	7,51	5,59	77,60	2,6	60	698	179
	202	300						63	603	152
	164	360						68	589	148
	112	420	148	7,455	5,97	76,10	4,5	78	603	152
**Hosgood**	118	0	149	7,535	6,66	81,50	11,4	72	1050	132
Group 3	182	60						101	1241	146
	127	120						152	852	139
	200	180						161	764	129
	224	240	155	7,647	5,17	74,10	10,4	194	548	116
	260	300						285	567	169
	296	360						252	376	153
	315	420	159	7,635	6,70	73,10	10,0	356	372	171
**Hosgood**	133	0	167	7,611	6,68	65,80	13,0	10	1779	172
Group 3	120	60						95	788	116
	41	120						136	698	129
	53	180						202	708	133
	88	240	139	7,336	4,73	55,30		256	610	150
	97	300						334	547	159
	126	360						506	466	150
	140	420	136	7,14	4,49	67,00	26,0	651	311	139
**Hosgood**	82	0	154	7,59	6,57	78,20	12,4	10	1833	165

Group 3	76	60						70	1254	146
	61	120						67	836	106
	76	180						87	820	116
	78	240	141	7,453	4,80	54,00	20,0	129	853	141
	94	300						207	803	151
	92	360						407	768	168
	96	420	141	7,299	4,90	65,00		638	564	143
**Hosgood**	72	0	165	7,708	6,31	66,70	13,0	5	1772	162
Group 3	82	60						77	912	142
	48	120						94	662	150
	71	180						148	554	148
	68	240	146	7,468	4,53	64,60	22,0	235	438	146
	63	300						449	474	>180
	84	360						583	331	154
	91	420	142	7,294	4,28	59,30	28,0	856	275	152
**Hosgood**	155	0	167	7,502	9,06	95,00	13,1	6	1681	152
Group 3	136	60						39	628	152
	126	120						53	418	123
	160	180						95	324	118
	179	240	136	7,213	4,52	74,60	23,0	119	202	109
	250	300						222	119	135
	260	360						478	138	142
	288	420	133	7,122	4,43	65,70	24,0	796	125	152
**MEPEP/Weissenb**	54	0	147	7,116	7,66	65,60	0,6	10	1148	159
Group 4	271	60						62	711	155
	287	120						125	612	160
	306	180						339	603	165
	292	240	155	7,431	5,23	59,70	3,6	638	580	169
	297	300						882	593	172
	275	360						998	574	172
	242	420	156	7,459	5,44	68,80	3,7	937	564	168
**MEPEP/Weissenb**	150	0	138	7,079	6,65	83,50	3,4	61	825	167
Group 4	853	60						135	423	159

	973	120						225	428	163
	965	180						377	401	165
	818	240	151	7,402	5,82	58,70	3,8	603	420	165
	700	300						833	420	167
	601	360						958	429	173
	399	420	154	7,397	5,96	56,10	4,4	983	395	172
**MEPEP/Weissenb**	172	0	150	7,107	9,10	62,70	0,4	23	1123	158
Group 4	648	60						52	598	155
	553	120						113	480	167
	509	180						153	488	175
	432	240	159	7,46	5,37	55,60	4,6	191	446	175
	388	300						228	433	177
	313	360						247	418	175
	257	420	162	7,505	5,49	69,40	4,5	264	432	179
**MEPEP/Weissenb**	111	0	149	7,206	6,62	78,90	0,8	9	1294	162
Group 4	322	60						51	491	161
	283	120						100	412	163
	240	180						176	416	170
	202	240	155	7,393	5,96	68,40	3,4	215	410	171
	235	300						263	402	175
	218	360						296	397	174
	218	420	157	7,432	6,10	73,60	3,2	313	410	171
**MEPEP/Weissenb**	80	0	141	7,24	6,48	71,10	0,4	12	904	154
Group 4	234	60						57	457	154
	202	120						81	453	156
	146	180						114	470	157
	144	240	142	7,291	5,91	60,80	5,1	166	458	158
	133	300						262	457	160
	119	360						379	427	151
	99	420	143	7,272	6,11	74,60	6,3	493	437	152

			Urine			
LDH (U/L)	TBARS	NAG	Creat (mmol/L)	Na (mmol/L)	Diuresis (ml)	Weight (grams)
135	11,76	1,32			0,0	307,39
247	6,79		1,4	129	11,0	
360	6,79	3,86	4,0	74	4,5	
469	7,25	6,23	4,7	58	6,0	
568	6,96	9,92	2,6	102	8,0	
615	8,36	12,77	0,8	151	29,0	
613	8,15	14,47	0,5	162	27,0	
631	8,70	17,77	0,4	166	22,5	427,64
119	8,46	1,11			0,0	284,87
246	5,96	1,93	1,8	118	42,5	
319	5,15	2,76	2,2	53	52,5	
420	6,17	3,03	1,5	102	27,5	
441	5,71	4,01	0,8	143	25,0	
535	6,18	4,09	0,5	139	39,0	
483	5,53	5,93	0,4	147	50,0	
510	5,93	8,80	0,4	150	79,0	452
185	6,59	1,70			0,0	267,79
223	5,27	1,71	0,5	144	60,0	
308	4,06	3,12	1,0	127	27,5	
348	5,16	4,88	0,8	135	43,0	
407	6,48	6,74	0,6	148	35,0	
437	5,34	9,68	0,4	154	33,0	
468	5,48	11,75	0,4	159	27,0	82,16+
501	5,84	16,88	0,4	162	19,5	349,95
226	5,44	1,27			0,0	280,26
567	4,18	2,22	1,4	131	27,0	
618	3,35	2,70	3,6	76	18,0	
642	5,00	2,99	3,9	66	15,0	
674	5,50	3,79	2,0	124	17,0	
666	4,86	5,01	0,6	158	30,0	
640	6,36	7,09	0,4	159	52,5	

618	6,49	10,24	0,4	161	52,5	450,54
122	6,39	0,90			0,0	324,72
265	5,21	2,01	0,7	146	45,0	
361	3,97	2,33	1,7	136	8,0	
459	3,56	3,09	1,1	130	13,0	
565	4,70	4,26	0,4	154	43,0	
531	4,31	5,82	0,4	159	73,0	
643	5,33	10,61	0,3	158	120,0	
595	5,55	13,98	0,2	162	158,0	644,1
208	1,91	0,38			0,0	286,06
288	1,14	3,05	1,0	120	11,0	
403	0,79	4,47	3,6	84	6,0	
498	1,44	4,26	4,1	60	1,0	
521	2,36	4,47			0,0	
622	2,49	5,82			0,0	
663	2,40	7,12	1,2	164	5,0	
706	2,59	9,02	0,8	146	2,0	341,7
120	1,98	1,33			0,0	303,45
332	1,15	1,77	0,7	153	3,5	
422	1,26	1,50	0,6	145	6,5	
498	2,09	1,36			0,0	
563	2,77	1,99			0,0	
619	2,76	2,39			0,0	
689	5,23	4,06			0,0	
770	5,12	7,27	9,4	183	9,0	391,86
102	1,86	0,71			0,0	324,3
232	1,34	2,56	2,0	113	10,0	
369	0,94	2,35	2,9	92	35,0	
421	1,69	2,24			0,0	
489	2,05	2,87			0,0	
687	2,34	3,15			0,0	
622	2,47	4,10			0,0	
733	2,01	6,59			0,0	396,26

48	1,36	1,04			0,0	248,97
208	0,87	2,48	3,6	95	33,0	
297	0,81	2,07	2,1	43	29,0	
447	1,92	2,70	4,5	67	8,0	
514	3,07	2,68	1,7	143	9,5	
486	5,27	3,41	0,5	174	8,0	
556	6,91	4,66	0,5	150	9,0	
607	7,41	6,73	0,5	183	17,0	342,98
468	3,45	3,09			0,0	230,44
291	2,44	2,18			0,0	
326	2,16	1,78			0,0	
337	2,52	1,64			0,0	
448	3,82	2,00			0,0	
410	5,49	1,44			0,0	
423	4,75	1,91			0,0	
453	5,55	2,61			0,0	249,19
256	1,16	4,90			0,0	279,82
362	1,64	6,00	0,4	124	220,0	
531	2,01	11,10	2,2	124	7,5	
559	1,52	18,20	1,3	122	5,0	
652	1,76	32,50	1,2	98	147,5	
907		33,20	1,1	114	126,0	
766	0,98	35,60	0,9	124	115,5	
875		47,50	0,6	124	130,0	501,78
56	2,43	0,50			0,0	265,47
429	2,67	5,30	1,4	161	135,0	
597	1,98	8,80	1,2	150	75,0	
804	1,86	15,00	1,0	145	60,0	
908	1,85	18,30	0,8	147	150,0	
951	1,78	21,70	0,6	143	125,0	
937	1,79	30,70	0,5	144	145,0	
906	1,70	42,10	0,4	144	150,0	383,22
39	1,82	0,50			0,0	221,89

333	2,36	2,60	1,7	158	12,5	
346	2,27	3,80	1,6	151	52,5	
432	2,40	5,80	1,4	148	60,0	
540	1,92	7,80	1,1	145	92,0	
613	2,42	11,00	1,0	151	100,0	
788	2,77	17,90	0,8	150	109,5	
785	1,97	33,20	0,6	151	100,0	370,3
18	2,44	0,70			0,0	282,3
434	1,42	3,80	1,5	157	175,0	
522	1,44	5,30	1,2	137	100,0	
704	1,13	9,10	0,9	135	125,0	
829	1,24	15,50	0,7	139	150,0	
940	1,94	15,80	0,5	143	100,0	
922	1,97	21,60	0,5	143	125,0	
1018	1,37	27,30	0,3	145	130,0	362
40	2,50	1,10			0,0	244,37
233	0,90	3,10	1,4	165	290,0	
298	0,69	4,90	0,8	145	230,0	
447	0,79	8,30	0,5	141	245,0	
350	0,71	7,90	0,4	144	250,0	
305		6,50	0,2	144	300,0	
523	0,62	9,50	0,1	143	175,0	
725	0,69	13,10	0,1	142	200,0	391,3
94	3,64				0,0	253,93
384	2,36	2,97	1,5	154	50,0	
572	2,26	6,06	1,5	108	65,0	
818	2,89	10,44	1,4	101	95,0	
1049	4,95	16,53	1,3	103	100,0	
1247	6,05	22,77	1,3	110	95,0	
1412	7,76	29,29	1,2	127	100,0	
1363	7,98	32,53	1,1	129	100,0	421,54
160	6,35				0,0	273,14
452	3,73	1,56	1	142	25,0	

685	3,76	6,17	1,3	109	35,0	
882	4,87	14,19	1,3	102	47,0	
975	6,09	29,60	1,1	97	22,5	
1119	9,89	25,87	0,9	107	29,0	
1284	11,29	24,09	1	118	18,0	
1302	5,94	27,85	0,9	141	22,5	359,31
108	1,40				0,0	281,3
290	2,01	1,64	2	165	33,0	
593	2,22	2,95	1,7	88	56,0	
623	1,98	4,21	1,8	80	58,0	
972	5,73	5,76	2	74	48,0	
1064	6,13	7,70	2,1	92	50,0	
1155	4,57	9,09	1,7	97	60,0	
1220	9,80	11,79	1,5	112	66,0	439,84
67	4,28	1,84			0,0	293,97
241	3,08	1,69	2	148	80,0	
378	2,74	4,33	2,4	90	77,0	
558	3,60	5,69	2,2	77	53,0	
681	4,61	6,85	2,4	81	50,0	
797	4,37	8,29	1,9	95	65,0	
869	7,09	8,90	1,6	112	80,0	
855	7,80	9,52	1,5	124	60,0	433,56
75	3,81				0,0	349,63
279	4,28	1,57	1,5	154	5,0	
414	3,52	2,15	0,9	140	17,0	
473	2,87	4,31	0,9	130	8,0	
554	11,09	6,81	0,9	131	6,0	
653	13,31	10,08	0,8	136	10,0	
724	14,06	15,76	0,7	139	10,0	
808	11,86	21,59	0,7	148	7,0	466,68
